# The Flexible Lubrication Performance of Graphene Used in Diamond Interface as a Solid Lubricant: First-Principles Calculations

**DOI:** 10.3390/nano9121784

**Published:** 2019-12-16

**Authors:** Jianjun Wang, Lin Li, Wentao Yang, Meng Li, Peng Guo, Bin Zhao, Linfeng Yang, Lili Fang, Bin Sun, Yu Jia

**Affiliations:** 1 Center for Quantum Functional Materials and Design, and College of Science, Zhongyuan University of Technology, Zhengzhou 450007, China; 2019003035@zut.edu.cn (W.Y.); limeng@zut.edu.cn (M.L.); goopoo@zut.edu.cn (P.G.); zhaobin@zut.edu.cn (B.Z.); 13938420289@163.com (L.Y.); hnzzfll@126.com (L.F.); sunbin7610@sina.com (B.S.); 2 Delivery Devices and Connected Solutions, Eli Lilly and Company, Indianapolis, IN 46285, USA; williamnism@hotmail.com; 3 International Laboratory for Quantum Functional Materials of Henan, and School of Physics and Engineering, Zhengzhou University, Zhengzhou 450001, China; jiayu@zzu.edu.cn

**Keywords:** graphene, diamond film, flexible lubrication, first-principles calculations

## Abstract

The interfacial friction performances of graphene covered and hydrogen-terminated diamond surfaces were investigated comparatively by first-principles calculations within density functional theory (DFT). Both systems exhibit similar excellent lubricating effects under small load, but the graphene covered interface presents small friction than that of hydrogenated system for the larger load. The calculated interfacial friction between two sheets of graphene covered diamond surface increases slowly than that of hydrogenated system in a wide range of pressure scale, and the friction difference between the two systems increases with increasing external pressure, indicating that graphene has flexible lubricating properties with high load-carrying capacity. This behavior can be attributed to the large interlayer space and a more uniform interlayer charge distribution of graphene covered diamond interface. Our investigations suggest that graphene is a promising candidate as solid lubricate used in diamond film, and are helpful for the understanding of interfacial friction properties of diamond film.

## 1. Introduction

Diamond coatings, from diamond single crystal (DSC) film to amorphous diamond-like carbon (DLC) film, have attracted great interest in both industrial application and scientific research fields due to its low friction, high wear resistance properties [1]. Sliding-induced graphitization [2,3,4,5] and passivation of dangling bond [6,7,8] are two widely accepted mechanisms for the excellent tribological behaviors of diamond films. Several researchers found that graphite-like carbon film formed by sliding-induced graphitization is the main reason for the low friction and wear of DLC films [3,4,5]. However, other experimental and theoretical studies supported the view that passivation of unsaturated carbon at the sliding interface is the main mechanism for the excellent friction properties [6,7,8]. Recent investigation reported that combined actions of graphitization and passivation are responsible for the appearance of ultra-low friction of diamond coatings [9]. However, as the complexity of the interface and the sensitivity of friction to external factors, such as pressure, humidity and sliding velocity, it is difficult to evaluate which mechanism plays a determining role in applications.

Compared to amorphous DLC films, DSC films have much simpler geometric structure, which is helpful for researchers to study contributions of special mechanism to complex friction phenomena of diamond coating [10]. As the simple crystal structure and the corresponding less calculation resource requirement, several theoretical calculations have investigated friction properties of DSC films and surfaces [8,11,12,13,14,15,16,17,18,19,20,21,22]. Because DSC films and surfaces are mainly composed of $sp^{3}$ bond, most of the calculations focus on the atomic passivation mechanism. These results show that terminated atoms and ion group, such as H, F, O and OH-, can effectively reduce interfacial activity, which corresponds to low friction and wear [8,11,12,13,14,15,16,17,18,19,20,21,22]. It also should be emphasized that the passivation effect is severely affected by the terminated atoms. For example, the friction in F atom terminated diamond surface is larger than that of H atom terminated system due to the larger filled electron density coverage from F atoms [8]. Sen et al., found that friction between two H- (resulting from the dissociative adsorption of $H_{2}$ molecules) passivated diamond surfaces is smaller than the interfacial friction between H- and OH- (resulting from the dissociative adsorption of $H_{2}O$ molecules) terminated structures [13]. More importantly, the friction behavior of terminated film or surface is also influenced by the load and passivation atom coverage [14,16]. So it is still difficult to accurately modulate the friction properties of DSC films and surfaces by atom surface passivation. Therefore, searching for ideal terminated materials and the improvement of lubricant effects are still important issues.

Due to its superior physical and chemical properties, graphene has been extensively studied in various fields in recent years [23]. Especially in the field of tribology, graphene has been considered as one of the most promise nanoscale lubricating material [24,25]. Different researchers have shown that graphene can be used as an excellent coating to reduce the surface and interface friction and wear of iron, bronze, steel, Ni, Re and Pt [26,27,28,29,30,31,32]. Recently, the interfacial properties of diamond film and graphene have also aroused much interesting in experimental and theoretical calculation researches [1,33,34]. As the perfect lattice matching between diamond (111) surface (C(111)) and graphene, several groups have prepared graphene in the C(111) surface successfully [1,33,34,35]. Theory and experimental works further studied the interfacial properties of graphene on C(111) surface, and revealed that weak van der Waals (vdW) interactions between graphene and diamond film dominate it’s the structures and properties [33,34,36,37,38,39]. Furthermore, several experimental studies have been carried on the friction properties of graphene on DLC and DSC films, where it was found that graphene can protect the surfaces and reduce its interfacial friction [40,41,42,43,44]. For example, Shen et al., investigated the friction of DLC film by using graphene as a solid lubricant [43]. Here it was shown that graphene could reduce the coefficient of friction (COF) of hydrogenated DLC film significantly. All the results indicate that graphene can be used as a coating to reduce friction and wear of diamond film. However, the detailed theoretical investigation of the interfacial friction behavior between graphene and diamond film is still limited, and its mechanism needs to be revealed.

To understand the lubricant mechanism of graphene on diamond film, the interfacial interaction and friction between graphene and C(111) surface were investigated by using first-principles calculations in this paper. Our calculations show that the graphene covered diamond interface exhibits a smaller friction than that of the hydrogenated one in a wide range of loads. The smaller friction can be attributed to large interlayer space and a more uniform interlayer charge distribution. We also found that graphene coating has flexible lubricating properties with high load-carrying capacity when used in the diamond film system.

## 2. Methods

The Vienna Ab-initio Simulation Package (VASP) code armed with the projector augmented-wave (PAW) method was used in our calculations [45,46]. The exchange–correlation interactions were handled with the generalized gradient approximation (GGA) in PBE [47]. The vdW interactions were added by the DFT-D2 approach, with a scaling factor $S_{6}=0.75$ [48]. An energy cutoff of 600 eV and Monkhorst-Pack grid for 2D irreducible Brillouin-Zone integration were chosen [49]. The convergence thresholds of total energy and Hellmann–Feynman forces were set to 10${}^{-5}$ eV and 0.01 eV/Å, respectively. A vacuum layer of about 20 Å was applied to shield the interactions between two adjacent cells.

To investigate the effects of graphene coating on tribological properties of diamond film, we first considered the interaction energy per unit area, $\Delta E=(E_{GD}-E_{G}-E_{D})/A$, where A is the area of calculation cell in optimized configuration, $E_{GD}$ is the total energy of graphene adsorbed on diamond film system, $E_{G}$ and $E_{D}$ are energy of isolated graphene and diamond film. By definition, the negative value of ΔE represents an attractive interaction between the two films. The work of separation $w_{sep}$, defined as the energy per unit area required to separate two films from contact to an infinite separation, can be estimated by the formula $w_{sep}=\Delta E$ [15]. By considering the interpolating and system symmetries, we can construct the potential energy surface (PES), $w_{sep}=\left(x,y,z_{eq}\right)$, which describes the variation of the $w_{sep}$ as a function of surface relative sliding position [15].

## 3. Results and Discussion

**Figure 1:**
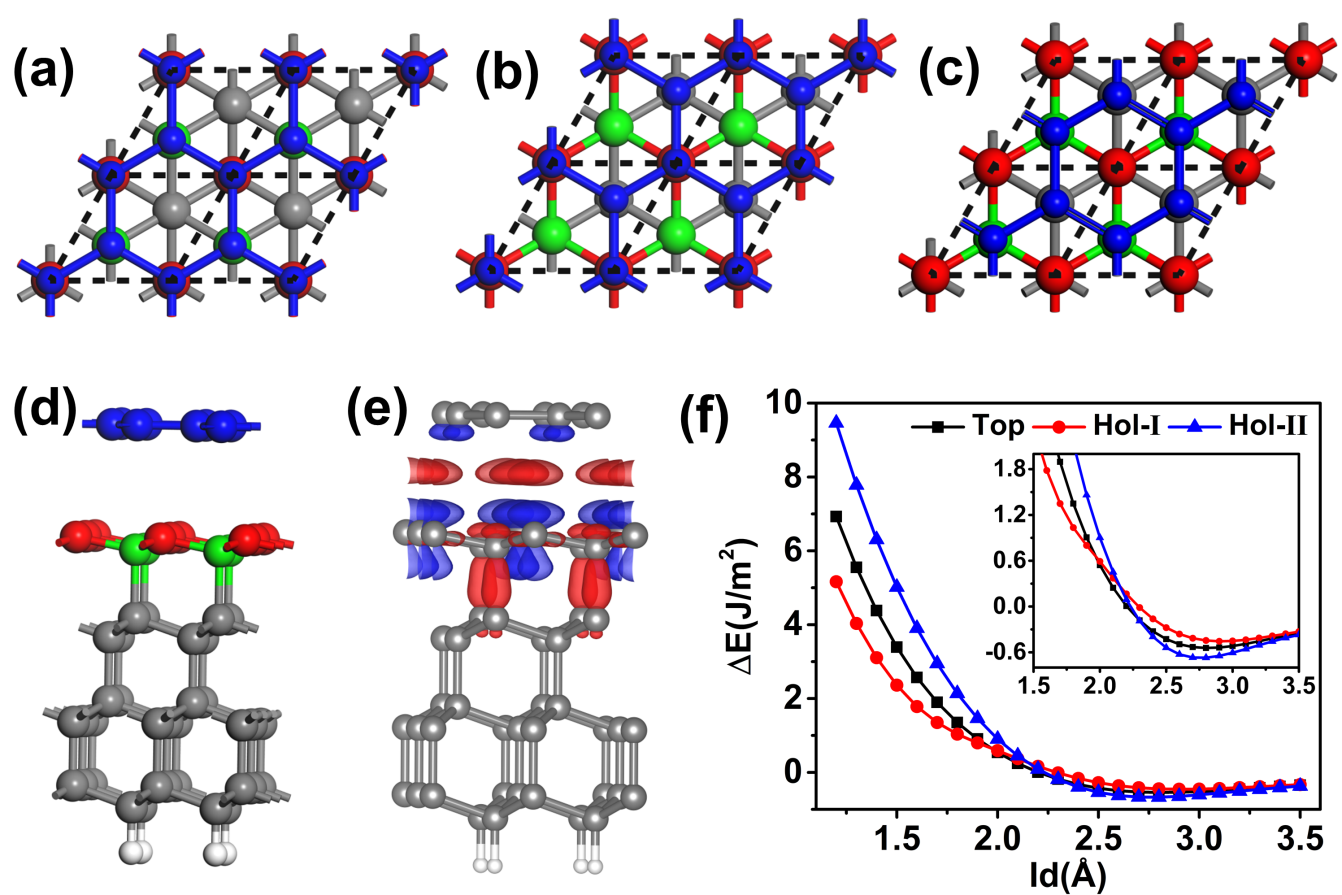
Top views of high-symmetry stackings of graphene-C(111) film: (**a**) Top, (**b**) hol-I and (**c**) hol-II. Side view of the hol-II stacking and its charge density difference are shown in (**d**,**e**), respectively. Blue balls represent C atoms in graphene; red and green balls represent C atoms in the first and second layers of C(111) film, and others C atoms and H atoms are labeled with grey and white colors. Red and blue represent increase and decrease of charge, and the isovalue is 0.0006 e/Å${}^{3}$. (**f**) The interaction energy as a function of interlayer distance (ld) between graphene and C(111) for the three stackings.

The existing experimental and theoretical results have confirmed that graphene can attach to the C(111) surface and form an interfacial structure [20,21,22,25]. More importantly, the lattice constant of graphene is very close with the basis vector length of the C(111) surface, which could reduce the super cell size of the calculating model. The optimized in-plane lattice parameter of C(111) and graphene were 2.46 and 2.45 Å, respectively, indicating the lattice mismatch between the two parts can be neglected. Therefore, we put the (1×1) graphene on (1×1) C(111) film to simulate the interface of graphene covered diamond film (Gra-C(111)), as shown in Figure 1a. The (1×1) C(111) film contains eight carbon layers, and the carbon atoms in the bottom layer were saturated by hydrogen atoms. In all calculations, all the atoms were free except for the C atoms in three bottom layers of C(111) film.

The stable structure of graphene adsorbing on C(111) film was calculated firstly. It should be noticed that the interlayer distance between the first and second layers is only 0.44 Å in the relaxed C(111) film, which approaches to one plane. The distance between the second and third layer was enlarged form 1.43 to 1.45 Å. The above results agreed well with the previous results that the diamond surface has a tendency of graphite. There are three high symmetry stackings for the Gra-C(111) structure. One is the top stacking, in which all of the C atoms in graphene located on the top of C toms in the first and second layers of C(111) film. Figure 1b,c exhibit two kinds of hollow stackings. In Figure 1b, half carbon atoms of graphene on the top C atoms in the first layer of C(111), and other half of C atoms on the top of C atoms located at the fourth layer of C(111) film. The stacking was defined as Hol-I. In Figure 1c, the C atoms in the second layer of C(111) surface are covered, and the C atoms in the first layer are located in the middle of the carbon ring of graphene, and the stacking was defined as Hol-II.

The interaction energy as a function of interlayer distance between graphene and C(111) film for the three stackings are shown in Figure 1f. From the figure, one can see that Hol-II stacking has the strongest interlayer adsorption interaction and Hol-I has the weakest one, which demonstrates that Hol-II is the most stable stacking. It should be noticed that the three curves intersect at 2.2 Å, indicating the stable sequence will take over around the interlayer distance. Our results agree well with the report that the interlayer interaction between graphene and DLC film belongs to vdW physical adsorption [37]. The stable stacking of Hol-II is shown in Figure 1e, and the charge density difference is also exhibited in Figure 1f. The charge transfer was around $10^{-4}$ e/Å${}^{3}$, which ensures that the interaction between the graphene and C(111) surface is physical adsorption.

**Figure 2:**
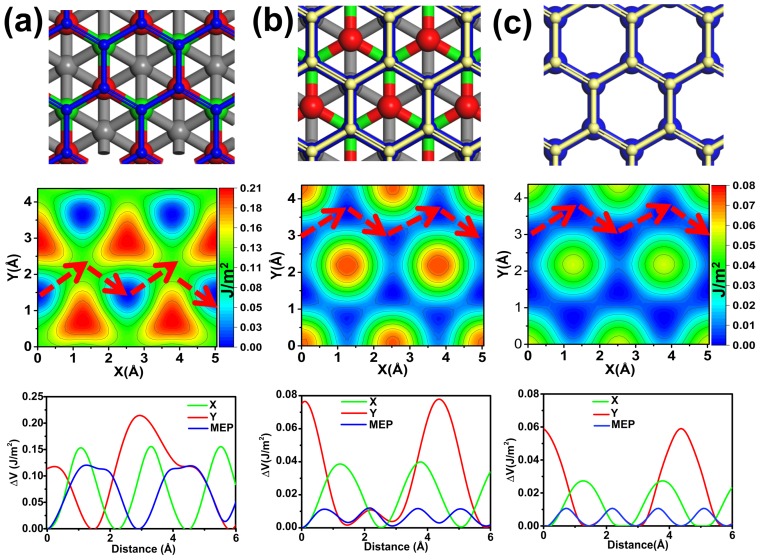
(**a**–**c**) Gra-C(111), graphene/Gra-C(111) and graphene/graphene systems. The top, middle and bottom planes are sliding model, Potential energy surfaces (PESs) and sliding barriers along special paths, respectively. Blue and yellow balls represent C atoms in graphene; red and green balls represent C atoms in the first and second layers of C(111) film, and others C atoms are labeled with grey color.

We next considered interfacial friction between graphene and C(111) film. Three kinds of sliding models were constructed to reflect the influence of diamond substrate on friction of graphene. For the first model, we put one layer of graphene on the C(111) substrate (Gra-C(111)) and then draw it across the diamond film substrate (Figure 2a). To reflect the shielding effect of graphene coating on C(111), we constructed a second model by adding another layer of graphene on top of the first model (graphene/Gra-C(111)), as shown in Figure 2b. Figure 2c shows the interfacial friction model of suspended bilayer graphene (graphene/graphene), which can be used as a reference for the first two structures. The middle panels of Figure 2 show the PESs of three structures. The interfacial friction difference between Gra-C(111) and graphene/graphene was compared firstly. The obvious character is that the Gra-C(111) system exhibits a larger potential wrinkle of about 0.21 J/m${}^{2}$, which is three times larger than that of the graphene/graphene systems. Besides wrinkle height, the morphologies of PES for the two systems are also very different. These results demonstrate that the interlayer interaction between graphene and C(111) surface are stronger than that of bilayer graphene.

Next, we explored the shielding effect of graphene on diamond film in the view of interfacial friction. From the comparison between Figure 2b,c one can see that the PES morphologies of Gra/Gra-C(111) and graphene/graphene are very similar, and the difference of wrinkle height of PES between the two systems is also unobvious. The sliding barriers along three special paths are given precisely, as shown in the bottom plane of Figure 2. The difference of wrinkle height of PES between Gra-C(111) and graphene/graphene systems is 0.15 J/m${}^{2}$, but the difference between the last two systems is only about 0.02 J/m${}^{2}$. These results demonstrate that the Gra-C(111) film exhibit larger and complex interfacial friction than that of graphene/graphene system, but diamond film substrate has an unobvious influence on the interfacial friction behavior of bilayer graphene, which indicate that graphene coating could provide good shielding and decrease the activity of diamond film effectively.

**Figure 3:**
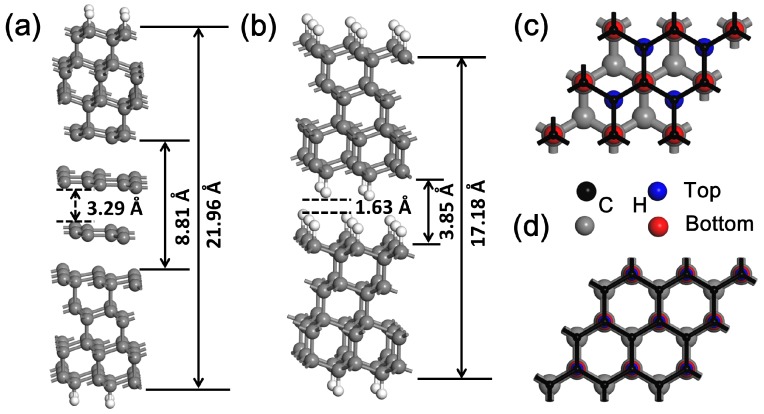
The sliding structures of (**a**) Gra-C(111)/Gra-C(111) and (**b**) H–C(111)/H–C(111) films. (**c**,**d**) are the interfacial structures of H–C(111)/H–C(111) system in hollow and top stackings. Black and blue balls represent C and H atoms in upper film, and grey and red balls represent C and H atoms in lower one.

In this part, the lubrication of graphene in the diamond interface was investigated. Two sheets of Gra-C(111) films were put against to simulate the role of reducing friction (Gra-C(111)/Gra-C(111)), as shown in Figure 3a. For the most stable structure, the middle two sheets of graphene stack in the form of hollow stacking. After structure relaxation, the interlayer distance between two graphene sheets is 3.29 Å, which is similar to the interlayer distance of bilayer graphene. To assessed the lubrication effect, hydrogenated diamond (111) film (H–C(111)) was chosen as a reference. Following our previous work, we constructed a sliding model of hydrogenated diamond film (H–C(111)/H–C(111)), as shown in Figure 3b [8]. Figure 3c,d shows the side views of the top and hollow stacking of the H–C(111)/H–C(111) system. The hollow stacking has the most strong adsorption energy, which is the most stable structure. For this stacking, the interlayer distance between the two slabs is only 1.63 Å, which is about a half of that in Gra-C(111)/Gra-C(111) systems.

**Figure 4:**
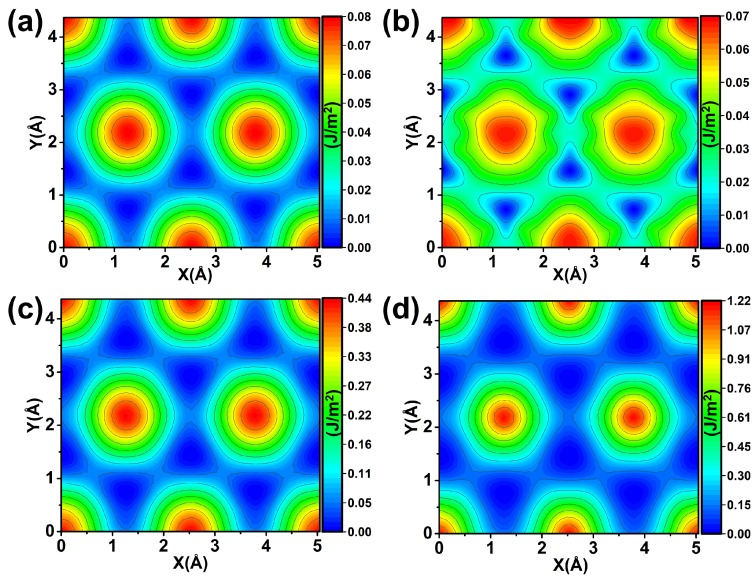
(**a**,**c**) The PESs of Gra-C(111)/Gra-C(111) system under loads of 0 and 20 GPa. (**b**,**d**) Are the cases of H–C(111)/H–C(111) system under loads of 0 and 20 GPa.

The PESs under free-load were constructed for the two systems, as shown in Figure 4a,b. Comparison between Figure 4a,b reveals that although the two systems exhibit different potential barrier shapes, the heights of potential wrinkles are almost equal. This result means that graphene and hydrogenation have a similar lubrication effect in the diamond surface under the situation of free-load. We then considered the load effect on the lubrication for the two systems. The 20 GPa pressure was applied on the two systems, and the corresponding PESs are shown in Figure 4c,d. From the figure, we can see that the two systems have similar morphology of PES but exhibited different relative barrier heights. The maximum sliding barrier is 1.22 J/m${}^{2}$ for H–C(111)/H–C(111) system, which is three times larger than that of Gra-C(111)/Gra-C(111) system. The results demonstrate that graphene coating exhibits better friction behavior than that of hydrogenation under loads.

**Figure 5:**
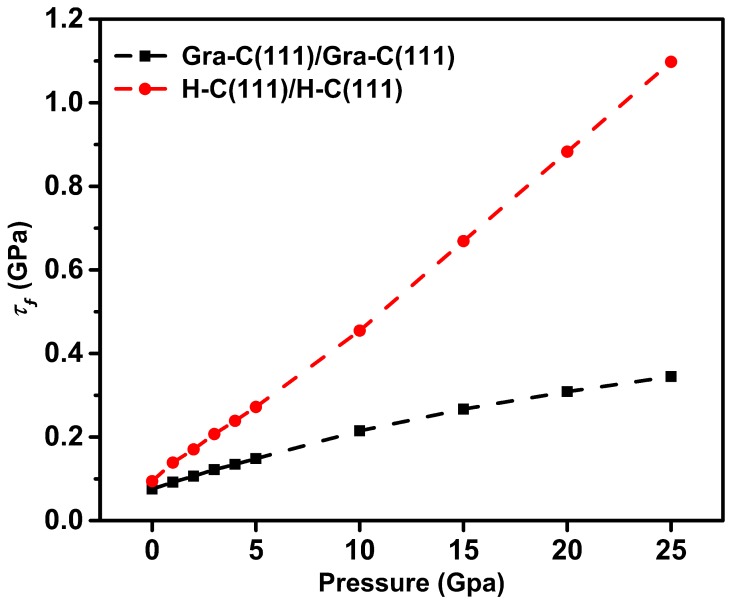
Comparison of shear strength $\tau_{f}$ between Gra-C(111)/Gra-C(111) and H–C(111)/H–C(111) interfaces under different normal pressures.

To reflect the effect of load on lubrication more directly, the shear strength $\tau_{f}$ along the high symmetry y directions was calculated (see Figure 4), in which the sliding barrier is the largest. The function relationship between $\tau_{f}$ and normal pressure are shown in Figure 5. From the figure, one can see that both systems have a similar $\tau_{f}$ in natural adsorption state. However, the gap of $\tau_{f}$ between the two systems increases with the increase of pressure. The $\tau_{f}$ in H–C(111)/H–C(111) system approximately linearly increases with the increase of pressure, but the increase is slight in the graphene covered system. The H–C(111)/H–C(111) system exhibits four times larger $\tau_{f}$ than Gra-C(111)/Gra-C(111) under the pressure of 25 Gpa, which is consistent with the experimental results [43]. These results illustrate that the lubrication property of graphene in the diamond film has a high load-carrying capacity. The weak response of friction to exert loads could be compared with the slow variation of elastic force to deformation in a soft flexible spring, and so we defined the behavior as flexible lubrication.

**Figure 6:**
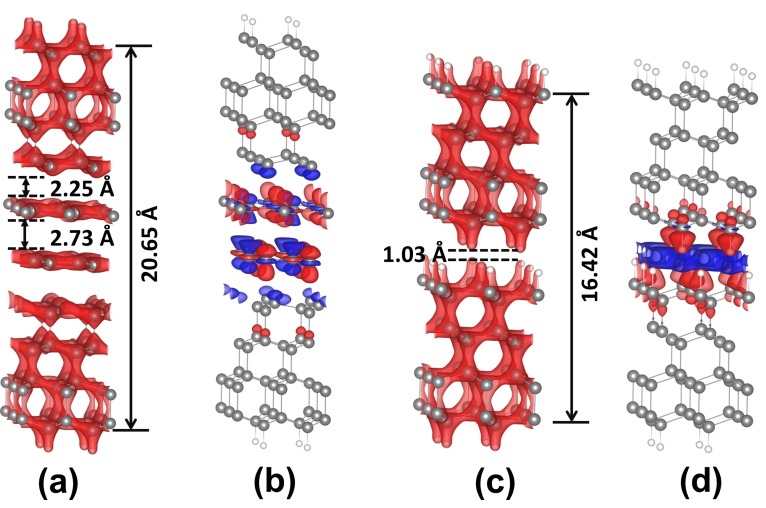
Charge structures. (**a**,**b**) Charge density distribution and charge density difference of Gra-C(111)/Gra-C(111) system under the load of 20 GPa. (**c**,**d**) The situation of H–C(111)/H–C(111) system.

To understand the lower friction behavior of Gra-C(111)/Gra-C(111) system, charge density distribution and charge density difference of the two systems were calculated, as shown in Figure 6. The interlayer distance between two graphene layers is 2.73 Å for Gra-C(111)/Gra-C(111) under the pressure of 20 GPa, which is larger than the interlayer space about 1 Å in H–C(111)/H–C(111) system. It is obvious that large interlayer separation can avoid interlayer interaction effectively. In Gra-C(111)/Gra-C(111) system, charge redistributes are located at the graphene sheet, and only have little change in interlayer space (Figure 6b), which contributes to a flat and smooth charge density distribution (Figure 6a). However, the charges rearrangement takes place in the interlayer region, and the accumulation of charge around the hydrogen atoms would cause a steep charge distribution at the interface of the H–C(111)/H–C(111) system. Therefore, the obvious difference in charge distribution between the two systems could explain the friction difference between them.

**Figure 7:**
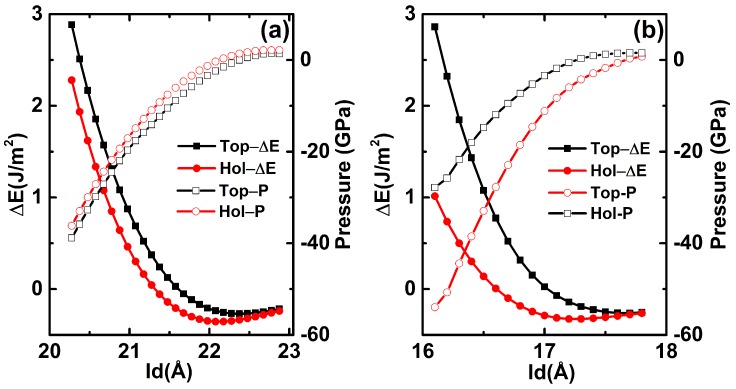
The interaction energy and pressure as a function of interlayer distance (IE). (**a**,**b**) Gra-C(111)/Gra-C(111) and H–C(111)/H–C(111) systems, respectively.

To further understand the reason why Gra-C(111)/Gra-C(111) system exhibits flexible lubrication, the dependence of interaction energy on interlayer distance was calculated, as shown in Figure 7. Figure 7a shows that when push the two Gra-C(111) films from 22 Å(the equilibrium distance between both ends of the system (see Figure 3a,b)) to 20 Å, the interaction energy becomes strong for both top and hollow stackings. However, the interaction energy difference between the two stackings at the same interlayer distance was kept. However, for the H–C(111)/H–C(111) system, the disparity of interaction energy between top and hollow stackings at the same distance becomes larger from 18 to 16 Å. As well known, the sliding barrier is mainly decided by the interaction energy difference. Therefore, as a load is applied, the interfacial friction in H–C(111)/H–C(111) will increase more quickly than that of Gra-C(111)/Gra-C(111) system. The dependence of normal load on interlayer distance can further confirm the above results. For Gra-C(111)/Gra-C(111) system, the interlayer distance decreases with the increase of normal load, but the difference of interlayer distance between the two stackings is very small and is almost unchanged throughout the whole applied load. Compared with Gra-C(111)/Gra-C(111) system, H–C(111)/H–C(111) system exhibits a larger difference of interlayer distance between the two stackings under same load. These results demonstrate that the sliding is more difficult in H–C(111)/H–C(111) than that of Gra-C(111)/Gra-C(111) system under the same loads. Figure 6 and Figure 7 illustrate the main flexible lubrication mechanism of graphene used in the diamond interface. The uniform charge distribution is responsible for the slight interaction energy difference between different stackings, and the sufficient interlayer space keeps the difference constant with load, which collectively determine the superior friction behavior in Gra-C(111)/Gra-C(111) system.

## 4. Conclusions

Based on the first-principles calculations, the interfacial friction properties of graphene on diamond surface have been investigated. Our results show that diamond film substrate has little influence on the interfacial friction properties of bilayer graphene, which indicates that the graphene can provide an effective shielding to the diamond surface. The friction properties between graphene covered diamond surface and hydrogenated terminated diamond surface have been investigated comparatively. Our results demonstrate that both systems exhibit similar small friction under the situation of free-load. However, with the increase of normal pressure, the sliding barrier in H–C(111)/H–C(111) system increases faster than that of Gra-C(111)/Gra-C(111) system, and the shear strength in H–C(111)/H–C(111) system is several times larger than that of later system. These low friction can be attributed to the large interlayer distance and more uniform interlayer charge distribution of graphene covered diamond interface. As diamond films are always used for devices operating in high-pressure environments, such as cutting tool, critical engine parts, artificial joints, and micro or nanoelectromechanical systems (MEMS/NEMS), this kind of flexible lubrication with high load capacity is meaningful for the practical application of diamond. This study suggests that graphene can be used in diamond film as a kind of excellent coating, and has a certain reference significance for the wear resistance modification of diamond film and surface.
